# Identification of patients with locally advanced rectal cancer eligible for neoadjuvant chemotherapy alone: Results of a retrospective study

**DOI:** 10.1002/cam4.6029

**Published:** 2023-05-06

**Authors:** Yi‐min Han, Wei‐xiang Qi, Shu‐bei Wang, Wei‐guo Cao, Jia‐yi Chen, Gang Cai

**Affiliations:** ^1^ Department of Radiation Oncology, Ruijin Hospital Shanghai Jiaotong University School of Medicine Shanghai China

**Keywords:** neoadjuvant chemoradiation, neoadjuvant chemotherapy, radiation therapy, rectal cancer

## Abstract

**Background and Objectives:**

Neoadjuvant chemotherapy (nCT) appears in a few clinical studies as an alternative to neoadjuvant chemoradiation (nCRT) in selected patients with locally advanced rectal cancer (LARC). We aimed to compare the clinical outcomes of nCT with or without nCRT in patients with LARC and to identify patients who may be suitable for nCT alone.

**Materials and Methods:**

A total of 155 patients with LARC who received neoadjuvant treatment (NT) were retrospectively analysed from January 2016 to June 2021. The patients were divided into two groups: nCRT (*n* = 101) and nCT (*n* = 54). More patients with locally advanced disease (cT4, cN+ and magnetic resonance imaging‐detected mesorectal fascia [mrMRF] positive [+]) were found in the nCRT group. Patients in the nCRT group received a dose of 50 Gy/25 Fx irradiation with concurrent capecitabine, and the median number of nCT cycles was two. In the nCT group, the median number of cycles was four.

**Results:**

The median follow‐up duration was 30 months. The pathologic complete response (pCR) rate in the nCRT group was significantly higher than that in the nCT group (17.5% vs. 5.6%, *p* = 0.047). A significant difference was observed in the locoregional recurrence rate (LRR); 6.9% in the nCRT group and 16.7% in the nCT group (*p* = 0.011). Among patients with initial mrMRF (+) status, the LRR in the nCRT group was significantly lower than that in the nCT group (6.1% vs. 20%, *p* = 0.007), but not in patients with initial mrMRF negative (−) (10.5% in each group, *p* = 0.647). Compared with the nCT group, a lower LRR was observed in patients in the nCRT group with initial mrMRF (+) converted to mrMRF (−) after NT (5.3% vs. 23%, *p* = 0.009). No significant difference was observed between the two groups regarding acute toxicity and overall and progression‐free survivals. Multivariate analysis showed that nCRT and ypN stage were independent prognostic factors for the development of LRR.

**Conclusion:**

Patients with initial mrMRF (−) may be suitable for nCT alone. However, patients with initial mrMRF (+) converted to mrMRF (−) after nCT are still at high risk of LRR, and radiotherapy is recommended. Prospective studies are required to confirm these findings.

## Graphical abstract

This study analyzed the relationship between the status of mesoretal fascia and local recurrence rate in patients with locally advanced rectal cancer after neoadjuvant therapy.

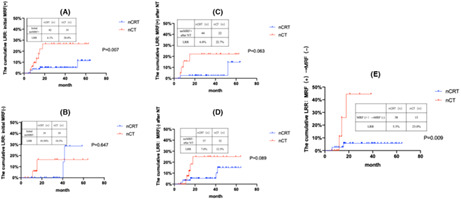

## 1 INTRODUCTION

Colorectal cancer is the third most common cancer in the world and the fifth most common cancer in China.^1^, ^2^ Neoadjuvant chemoradiotherapy (nCRT) is the standard treatment modality for Stage II/III rectal cancer.^3^ In patients with locally advanced rectal cancer (LARC), (total) neoadjuvant therapy is preferred if the circumferential resection margin (CRM) is negative and highly recommended if positive. Although there are numerous trials on nCRT combined with medication therapy, 5‐FU or capecitabine alone is suggested at present.^3^, ^4^, ^5^

A phase II trial by Ishii showed that nCT comprising a combination of drugs, such as irinotecan and fluorouracil with leucovorin, resulted in an acceptable down‐staging effect.^6^ The multicentre phase III FOWARC trial showed no significant difference in the 3‐year disease‐free survival (DFS) or locoregional recurrence rate (LRR) with perioperative mFOLFOX6 with or without radiation plus fluorouracil.^7^ Based on these results, nCT alone has become a potential therapeutic strategy for selected patients. According to the National Comprehensive Cancer Network (NCCN) guidelines,^8^ nCT of FOLFOX or capecitabine plus oxaliplatin alone could be an option for pT3N0M0 margin‐negative tumours, high in the rectum or at the rectosigmoid junction. However, many clinical problems associated with local recurrence have not been resolved. The choice of nCRT or nCT alone remains a research focus in the field of rectal cancer.

Clinically, the involvement of mesorectal fascia (MRF) is a prognostic factor for local recurrence.^9^ with a sensitivity and specificity of up to 94% and 76%, respectively, magnetic resonance imaging (MRI) is particularly accurate in estimating the distance between the tumour and the MRF.^10^ The assessment of MRI‐MRF before surgery plays a key role in LRR. The relevant research demonstrated that the ADC value could predict response of rectal tumour to nCRT.^11^ Besides, dynamic contrast‐enhanced MRI parameters such as *Wash*‐*out* and *K*_ep_ could predict tumour aggressiveness and nCRT efficacy.^12^ According to the MERCURY study, the local recurrence hazared ratio for MRI‐involved MRF was 3.50 (95% CI, 1.53–8.00; *p* < 0.05).^13^ However, the proportion and risk factors for developing LRR among LARC patients with varying MRF status treated with nCT alone remain unknown. In addition, data regarding whether nCT alone can be used in patients with initial magnetic resonance imaging‐detected mesorectal fascia (mrMRF) (+) converted to mrMRF (−) after nCT is limited. This study aimed to compare the therapeutic outcomes in patients with LARC who received nCT or nCRT and to define a potential subgroup of these patients who might be candidates for nCT alone.

## 2 PATIENTS AND METHODS

### 2.1 Patient population

This retrospective clinical study analysed 155 patients with LARC who were treated between January 2016 and June 2021 at a single centre. The inclusion criteria were as follows: (1) histologically confirmed rectal cancer with no clinical evidence of distant metastasis, (2) Stage II or III cancer evaluated by computed tomography and MRI in accordance with the eighth edition of the American Joint Committee on Cancer, and (3) those receiving nCRT or nCT followed by total mesorectal excision (TME) surgery. The exclusion criteria were as follows: (1) special histological types such as neuroendocrine carcinoma and melanoma. (2) Lack of baseline MRI data and (3) a stage IV initial diagnosis. In total, 250 patients were retrospectively enrolled.

### 2.2 Study period

The study period was from 1 August 2020 to 22 March 2022.

### 2.3 Treatment method

Both groups received a standard CAPEOX or FOLFOX regimen as nCT. In the nCRT group, nCT was delivered in two (range, 1–8) cycles before and after radiotherapy. The median number of nCT cycles in the nCT group was four (range, 2–6). Capecitabine 825 mg/m^2^ bid daily 5 days/week was delivered concurrently with radiotherapy in the nCRT group. The irradiation technique was performed using a linear accelerator with a 6 MV photon beam‐based intensity‐modulated radiation therapy or volumetric modulated arc therapy. The delineation of the clinical target volume was referred to as the Radiation Therapy Oncology Group atlas,^14^ which included the primary tumour and at‐risk nodal areas. The irradiation regimen consisted of 50  Gy in 25 fractions, administered for 5 weeks. Surgery was planned within 12  weeks (median 8.9 weeks, range 6–20 weeks) after radiotherapy in the nCRT group. Adjuvant chemotherapy was delivered in 4 (range, 2–7) cycles in the nCRT group and 3 (range, 1–6) cycles in the nCT group. The median cycle of chemotherapy (including neoadujvant and adjuvant chemotherapy) in the nCRT group was 5 (range 1–8 cycles), while in the nCT group was 6 cycles (range 3–8 cycles). Thirteen patients in the nCT group received adjuvant radiotherapy.

### 2.4 Data collection

All patients' clinical records, including MRI images, neoadjuvant treatment (NT), clinicopathological characteristics, adjuvant treatment, recurrence and survival information, were retrospectively reviewed. Tumour node metastasis classification and tumour regression grade (TRG) were in accordance with the American Joint Committee on Cancer/International Union for Cancer Control (eighth edition).^15^ The mrMRF images were assessed by two senior radiologists. Adverse events were described using the National Cancer Institute Common Terminology Criteria (version 3.0). Overall survival was defined as the time interval between the date of diagnosis and the date of death or the latest follow‐up date. DFS was defined as the interval between the date of NT and the date of the first recurrence, last follow‐up, or death, whichever occurred first.

### 2.5 Statistical analysis

The chi‐square test or Fisher's exact test was used to compare clinical information, chemotherapy regimens, acute toxicity of the preoperative regimens and pathologic complete response (pCR) rates between the two groups. Variable Kaplan–Meier survival curves were used to compare recurrence events, OS and DFS. Univariate and multivariate logistic regressions were performed to investigate independent factors predicting the achievement of locoregional recurrence‐free survival. Statistical significance was defined at *p* < 0.05. All *p*‐values were two‐sided. Statistical analyses were performed using Prism (version 9.0; GraphPad Software).

To reduce bias of cT stage, cN stage and initial mrMRF, we conducted a propensity score matching analysis. The propensity score was calculated by using a logistic regression model including cT stage, cN stage and mrMRF. We then formed matched pairs between nCT and nCRT group patients using the nearest neighbour matching method with a calliper of 0.03, and one to one matching algorithm was performed within default calliper (0.03) in R version 3.4.2 software (The R Foundation for Statistical Computing http://www.r‐project.org).

## 3 RESULTS

### 3.1 Baseline characteristics

Table 1 presents the baseline characteristics of the patients enrolled in this study. In total, 101 and 54 patients received nCRT and nCT, respectively. Compared with the nCT group, patients in the nCRT group presented with more locally advanced diseases, such as the cT4 stage, cN stage and initial mrMRF (+). Lesions greater in the rectum (i.e. a length of >10 cm between the tumour and the anal verge) were observed in the nCT group.

**TABLE 1:** Clinical characteristics of 155 patients with rectal cancer.

	nCRT group (*n* = 101)	nCT group (*n* = 54)	*p‐*value
Sex, *n* (%)			0.623
Male	73 (72.3)	41 (75.9)	
Female	28 (27.7)	13 (24.1)	
Age, median year (range)	68 (range 31–75)	60 (range 34–75)	
AJCC Stage, *n* (%)			
II	7 (6.9)	7 (13.0)	0.211
III	94 (93.1)	47 (87.0)	
cT stage, *n* (%)			0.003
T3	40 (39.6)	35 (64.8)	
T4	61 (60.4)	19 (35.2)	
cN stage, *n* (%)			0.043
N0	7 (6.9)	7 (13.0)	
N1	27 (26.7)	20 (37.0)	
N2	67 (66.3)	27 (50.0)	
Distance of tumour from the anal verge, *n* (%)			0.059
0–5 cm	41 (40.6)	22 (40.7)	
6–10 cm	40 (39.6)	7 (13.0)	
>10 cm	20 (19.8)	25 (46.3)	
ECOG Performance Status, *n* (%)			
0	7 (6.9)	5 (9.3)	0.605
1	94 (93.1)	49 (90.7)	
Histologic type, *n* (%)			0.495
Adenocarcinoma	93 (92.1)	52 (96.3)	
Mucinous adenocarcinoma	8 (7.9)	2 (3.7)	
mrMRF, *n* (%)			0.031
+	82 (81.2)	35 (64.8)	
−	19 (18.8)	19 (35.2)	
nCT cycle, median (range)	2 (1–8)	4 (2–6)	0.013

Abbreviations: AJCC, American Joint Committee on Cancer; ECOG, Eastern Cooperative Oncology Group.

### 3.2 Surgery and pathology

No differences were observed in the surgical procedures. The overall sphincter preservation rate was 67.7%, and there was no significant difference between the nCRT and nCT groups. The pCR rate was significantly higher in the nCRT group than in the nCT group (17.5% vs. 5.6%, *p* = 0.047). In addition, more patients in the nCT group presented with TRG 3 than those in the nCRT group (40.7% vs. 2.9%, *p* < 0.001). A higher proportion of poor‐differentiation was observed in the nCT group (10.8% vs. 31.5%, *p* < 0.001). Patients in the nCRT group had a significantly higher ypN0 stage rate than those in the nCT group (70.3% vs. 33.3%, *p* < 0.001). The surgical and pathological information is listed in Table 2.

**TABLE 2:** Surgical procedures and pathologic staging.

	nCRT group (*n* = 101)	nCT group (*n* = 54)	*p*
Type of surgery, *n* (%)			0.556
Anterior resection	66 (65.3)	38 (70.3)	
Abdominoperineal resection	33 (32.7)	15 (27.8)	
Other	29 (2.0)	1 (1.9)	
Sphincter preservation, *n* (%)	67 (66.3)	38 (70.3)	0.609
TRG score, *n* (%)			<0.001
0	18 (17.8)	3 (5.6)	
1	36 (35.6)	5 (9.3)	
2	44 (43.6)	24 (44.4)	
3	3 (3.0)	22 (40.7)	
pCR, *n* (%)	18 (17.8)	3 (5.6)	0.047
Pathological differentiation, *n* (%)			<0.001
Well‐differentiation	9 (8.9)	3 (5.6)	
Moderate differentiation	63 (62.4)	31 (57.4)	
Poor differentiation	11 (10.8)	17 (31.5)	
Neural invasion, *n* (%)	11 (10.9)	7 (13.0)	0.703
EMVI, *n* (%)	12 (11.9)	5 (9.3)	0.619
ypT stage			0.070
T0–2	47 (46.5)	17 (31.5)	
T3–4	54 (53.5)	37 (68.5)	
yN stage			<0.001
N0	79 (70.3)	18 (33.3)	
N1–2	22 (28.7)	36 (66.7)	

Abbreviations: EMVI, extramural venous invasion; pCR, pathologic complete response; TRG, tumour regression grade.

### 3.3 Toxicities

Table 3 shows the incidence of acute toxicity during NT. No statistical differences were observed in any adverse events or Grade ≥3 toxicities between the groups. The most common Grade 3–4 toxicities were leukopenia and radiation‐related dermatitis. Compared with the nCRT group, significantly higher Grade 1–2 levels of thrombocytopenia (3.0% vs. 29.6%) and Grade 3–4 levels of leukopenia (1.0% vs. 9.3%) were observed in the nCT group. Regarding non‐haematological toxicity, more patients experienced nausea in the nCT group (9.9% vs. 27.8%). Meanwhile, the frequency of radiation dermatitis was significantly higher in patients in the nCRT group.

**TABLE 3:** Acute toxicity of neoadjuvant treatment.

	All grades, *n* (%)			Grade 3 or 4, *n* (%)		
	nCRT group (*n* = 101)	nCT group (*n* = 54)	*p*	nCRT group (*n* = 101)	nCT group (*n* = 54)	*p*
Any adverse event	75 (74.3%)	47 (87.0%)	0.064	19 (18.8%)	8 (14.8%)	0.532
Leukopenia	57 (56.4%)	36 (66.7%)	0.220	1 (1.0%)	5 (9.3%)	0.020
Anaemia	7 (6.9%)	2 (3.7%)	0.497	0 (0%)	0 (0%)	NS
Thrombocytopenia	3 (3.0%)	16 (29.6%)	<0.001	0 (0%)	0 (0%)	NS
Diarrhoea	55 (54.5%)	10 (18.5%)	0.035	8 (7.9%)	1 (1.9%)	0.163
Nausea	10 (9.9%)	15 (27.8%)	0.006	1 (1.0%)	2 (3.7%)	0.290
Vomiting	16 (15.8%)	10 (18.5%)	0.673	0 (0%)	0 (0%)	NS
Radiation dermatitis	32 (31.7%)	‐	NS	9 (8.9%)	‐	NS
Cystitis	8 (7.9%)	‐	NS	0 (0%)	‐	NS

### 3.4 Survival outcomes

The median follow‐up period was 30 months. The 2‐year DFS (79.2% vs. 75.9%, *p* = 0.752) and OS (89.1% vs. 94.4%, *p* = 0.866) were comparable between the nCRT and nCT groups, respectively. The cumulative total recurrence rate did not differ between groups. A significantly lower LRR rate was observed in the nCRT group (6.9% vs. 16.7%, *p* = 0.011; Table 4). The failure pattern of LRR in the nCRT group was observed in three cases with anastomotic recurrence, three with pelvic lymph node metastasis, and one with a pelvic mass. In the nCT group, six patients developed pelvic lymph node metastasis and three patients experienced anastomotic recurrence and pelvic mass. There was no significant difference in the number of patients with distant metastases (*p* = 0.752).

**TABLE 4:** Recurrence information during follow‐up.

Pattern of recurrence	nCRT group (*n* = 101)	nCT group (*n* = 54)	*p*	RT group (*n* = 114)	Non‐RT group (*n* = 41)	*p*
Locoregional recurrence LRR	7 (6.9%)	9 (16.7%)	0.011	8 (7.0%)	8 (19.5%)	0.040
Distance metastasis	22 (21.8%)	10 (18.5%)	0.752	23(20.2%)	9 (22.0%)	0.303
Total recurrence	27 (26.7%)	13 (24.1%)	0.848	28 (24.6%)	12 (29.3%)	0.555

Abbreviations: Non‐RT, non‐radiation group; RT, radiation group.

*Note*: (1) Thirteen patients received adjuvant radiotherapy in the nCT group. (2) Two and six patients had both locoregional recurrence and distant metastasis in the nCRT and nCT groups, respectively.

Thirteen patients in the nCT group received adjuvant radiotherapy, and all patients were regrouped according to treatment with perioperative pelvic radiation. A total of 114 (73.5%) patients received radiotherapy between treatments, whereas 41 (26.5%) did not. Table 4 illustrates the significant differences in LRR rates between the radiation and non‐radiation groups (*p* = 0.040).

### 3.5 Correlation between MRF status and LRR

**FIGURE 1:**
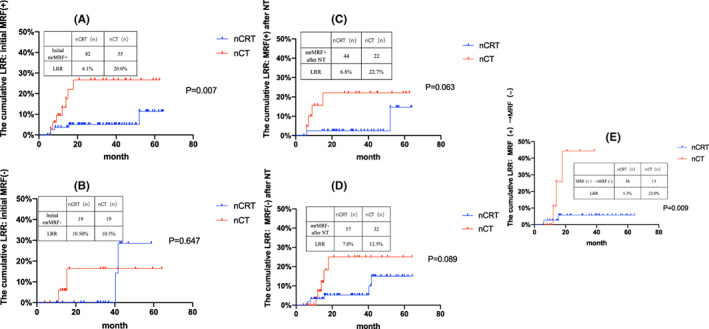
A‐1E: The relationship between MRF status and LRR. Cumulative incidence of LRR in patients with; 1A initial mrMRF (+); 1B with initial mrMRF (−); 1C mrMRF (+) after NT; 1D mrMRF (−) after NT; 1E initial mrMRF (+) were converted to mrMRF (−) post NT.

Of all the patients, 82 and 35 were confirmed to have initial mrMRF (+) status before treatment in the nCRT and nCT groups, respectively. After NT, 44 patients in the nCRT group and 22 in the nCT group were consistently confirmed to be mrMRF (+). The cumulative LRR in patients with initial mrMRF (+) status was 6.1% (5/82) in the nCRT group and 20% (7/35) in the nCT group (log‐rank *p* = 0.007; Figure 1A). For patients with mrMRF (+) status after NT, the LRR rate was 6.8% (3/44) in the nCRT group and 22.7% (5/22) in the nCT group (*p* = 0.063; Figure 1C). In both groups, patients with initial mrMRF (−) had the same rate of LRR (10.5%, *p* = 0.647; Figure 1B). The rate of LRR in patients with mrMRF (−) after nCT in the nCT group was 12.5%, and no difference in LRR was observed after NT (*p* = 0.089; Figure 1D). Further analyses showed that 46.3% (38/82) of patients with initial mrMRF (+) were converted to mrMRF (−) post nCRT; 37.1% (13/35) of patients were similarly converted in the nCT group. In patients whose mrMRF status changed, the LRR rate was significantly lower in the nCRT group (5.3% vs. 23.1%, *p* = 0.009; Figure 1E).

### 3.6 Risk factor analysis

In univariate analyses of demographics, clinical stage and histological features of biopsy specimens, nCRT and ypN stage were risk factors for LRR. In multivariate analyses, the ypN2 stage and nCT were associated with a higher LRR. The findings from the univariate and multivariate analyses of LRR are described in Table 5.

**TABLE 5:** Univariate and multivariate analysis for LRR.

Factors	Univariate analysis				Multivariate analysis			
	*p*	HR	95%CI		*p*	HR	95%CI	
			Lower	Upper			Lower	Upper
LRR								
Age								
<65	Ref.	1						
≥65	0.766	0.860	0.319	2.317				
Sex								
Men	Ref.	1						
Women	0.621	1.306	0.453	3.766				
Type of NT								
nCT	Ref.	1						
nCRT	0.02	0.307	0.114	0.827	0.025	0.305	0.108	0.859
ypN stage	0.014				0.008			
0	Ref.	1			Ref	1		
1	0.019	3.395	1.226	9402	0.03	3.1	1.119	8.588
2	0.024	11.656	1.378	98.567	0.01	17.696	1.965	159.322
cT stage								
cT3	Ref.	1						
cT4	0.329	0.625	0.243	1.606				
cN stage	0.494							
N0	Ref.	1						
N1	0.329	2.887	0.344	24.210				
N2	0.597	1.747	0.221	13.801				
Distance of tumour from the anal verge	0.186							
0–5 cm	Ref.	1						
6–10 cm	0.339	0.596	0.206	1.721				
>10 cm	0.075	0.245	0.245	1.155				

Abbreviation: LRR, local recurrence rate.

### 3.7 Propensity score‐matched analysis

**FIGURE 2:**
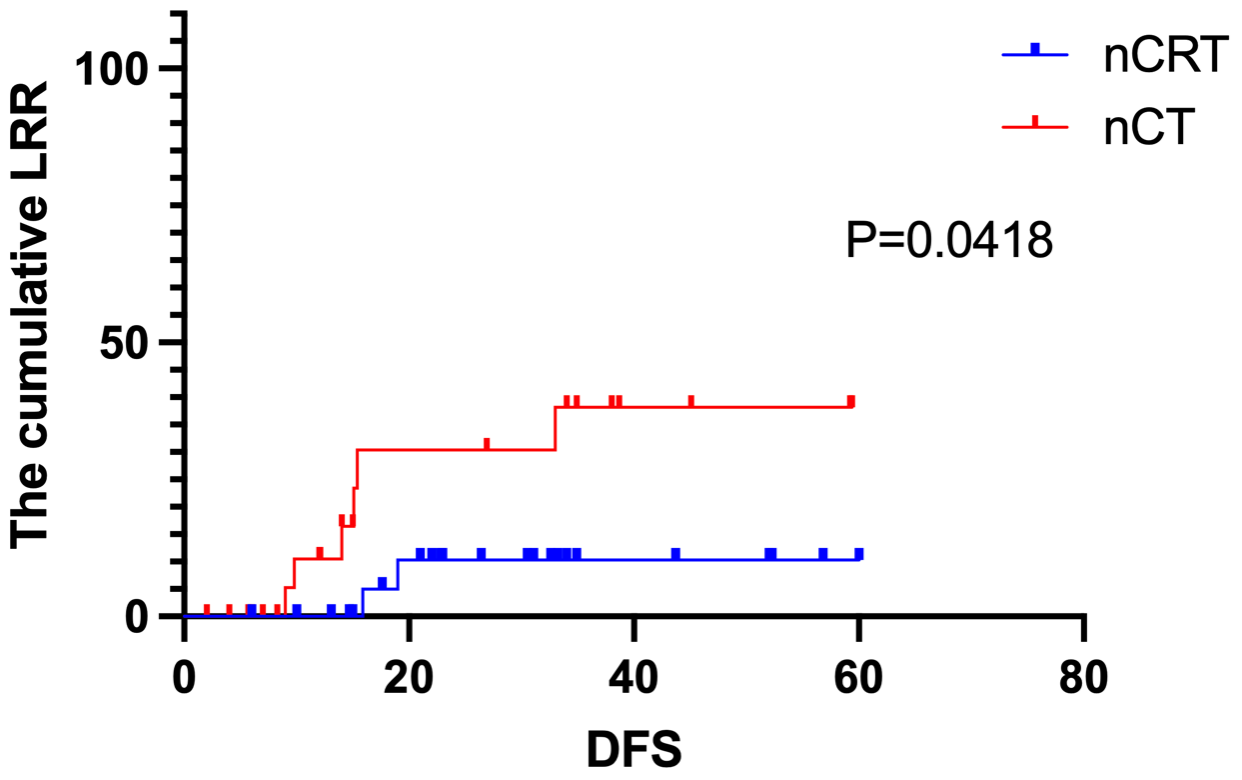
LRR of 50 patients with propensity score‐matched analysis.

Through the propensity matching analysis, 50 cases were successfully matched, including 25 cases in the nCRT group and 25 cases in the nCT group. The median follow‐up time was 33.2 months. The cumulative LRR between the two groups was 2/25(8%) in the nCRT group and (6/25)24% in the nCT group (*p* = 0.0418, Figure 2), the clinical characteristics of 50 patients after propensity score‐matched analysis was listed in Table 6.

**TABLE 6:** Clinical characteristics of 50 patients with propensity score‐matched analysis.

	nCRT group *n* = 25	nCT group *n* = 25	*T*/*χ* ^*2*^	*p*
Sex, *n*			0.117	0.733
Male	19	20		
Female	6	5		
Age (average ± SD)	54.7 ± 2.5	60.7 ± 2.0	0.252	0.066
cT, *n*			0	NS
T3	13	13		
T4	12	12		
cN, *n*			0	NS
0	7	7		
1	18	18		
Distance, *n*				
≥5 cm	12	18	3	0.083
<5 cm	13	7		
Initial MRF, *n*			0.5	0.480
Positive	6	4		
Negaitve	19	21		

## 4 DISCUSSION

(Total) nCRT is currently the standard treatment for LARC.^16^, ^17^, ^18^, ^19^, ^20^ However, nCT alone has been occasionally used by certain surgeons because of the increased difficulty of surgical operations caused by radiotherapy. The NCCN guidelines do not mention whether radiation should be administered to patients with mrMRF status changes after NT.^8^ The LRR rate of the initial mrMRF (+) status converted to mrMRF (−) after nCT remains unclear. Therefore, we retrospectively analysed relevant data from our centre. Based on the results of our analysis, patients treated with nCT alone had a poorer pCR rate and higher LRR than those treated with nCRT. Patients with initial mrMRF (−) may be eligible for nCT alone. Patients with initial mrMRF (+) converted to mrMRF (−) after nCT are still at high risk for LRR, and radiotherapy is recommended.

Consistent with previous results of randomised controlled clinical studies, the pCR rate and TRG 0–1 were much higher in the nCRT group.^4^, ^18^, ^21^, ^22^ A higher proportion of TRG 0–1 was found in the nCRT group than in the nCT group, which indicated that nCRT had a positive effect on tumour regression in LARC. Generally, the pCR rate of patients with LARC who receive nCT is <10%.^23^ Despite the CONVERT trial's suggestion that nCT alone can achieve an 11% pCR rate, patients enrolled in the nCT arm had a low burden.^24^ Another phase II trial treated patients with mFOLFOXIRI for five cycles as a neoadjuvant regimen.^25^ The pCR rate was only 4.3%. The objective of modern treatment for LARC is organ preservation, particularly in patients with lower rectal cancer. According to reported data, pCR rate and TRG are associated with organ preservation.^26^ Therefore, the addition of radiotherapy during NT can increase the pCR and organ preservation rates.

LRR rates of 5%–10% have been reported in other studies following nCRT.^27^, ^28^, ^29^, ^30^ Notably, the baselines of the two groups in our study were not balanced. Although more patients with locally advanced diseases were observed in the nCRT group than in the nCT group, a higher pCR rate and lower LRR were achieved in the nCRT group. Afterwards, we performed propensity score‐matched analysis to eliminate the initial imbalance between cT stage and cN stage. The LRR rate was still significantly lower in the nCRT group (8% vs 24%, *p* = 0.0418). Multivariate analysis revealed that nCRT or nCT and ypN stage were independent factors for LRR. Patients with the ypN2 stage had nearly 17 times higher odds of LRR than those with the ypN0 stage. The most common local failure pattern in the nCT group was pelvic lymph node metastasis (6/9, 66%), which appeared at a significantly higher rate than in the nCRT group (3/7, 42.9%). Therefore, according to the results of the multivariate analysis and local failure pattern, patients with the ypN2 stage have a high risk of LRR. In such patients, standard treatment may be insufficient. Therefore, more aggressive treatment may be required, and specific treatment regimens should be developed.

MRF is considered an important predictor of LRR and may help identify patients for preoperative radiotherapy.^31^ In the RAPIDO trial, 60% of patients in the nCRT group had baseline MRF (+), and 6% had local relapse.^32^ Our data suggest that 81.2% of patients with initial MRF (+) in the nCRT group benefited from loco‐regional radiation (LRR 6.1% in nCRT vs. 20% in nCT, *p* = 0.007). However, no statistical difference was observed in LRR in patients with an initial mrMRF (−). Another study revealed that mrMRF (+) post‐NT was an independent risk factor for LRR, OS and DFS.^33^ This result emphasises the need to add radiotherapy to patients with initial MRF (+) and may select nCT alone for those with initial MRF (−). In addition, no available data have elucidated whether radiotherapy is necessary for patients with MRF (+) who have successfully converted to MRF (−) after nCT. Compared with the nCT group, a significant reduction in LRR was observed in patients with initial mrMRF (+) converted to mrMRF (−) in the nCRT group (5.3% vs. 23%, *p* < 0.01). This indicates that nCRT is still required for patients with an initial mrMRF (+) converted to mrMRF (−) after nCT. Prospective studies (e.g. the PROSPECT and NCT02288) of nCT without routine radiation in selected patients may determine the effects of nCT alone and provide more clinical evidence.

In this study, the adverse effects of neoadjuvant therapy were acceptable. The overall incidence of Grade 3–4 toxicity was <20%. The rate of radiotherapy‐related adverse reactions was similar to those reported in other clinical studies.^34^, ^35^ High haematological toxicities (any grade of thrombocytopenia and Grade 3–4 leukopenia) were observed in the nCT group.

The strength of this study is that it explores some treatment principles that are not included in the current clinical guidelines through retrospective analysis and makes up for the blank in this regard through data analysis. The limitations of this study were as follows: this was a single‐centre, retrospective analysis with small sample size; therefore, potential selection bias could not be avoided. Adjuvant chemotherapy of the two groups might be not balanced. There were 4 (range, 2–7) cycles in the nCRT group and 3 (range, 1–6) cycles in the nCT group. It is debatable to administer additional adjuvant chemotherapy in patients with Stage II/III rectal cancer who received nCRT.^36^, ^37^ However, there was no statistically significant difference in the total cycles of chemotherapy (neoadujvant and adjuvant chemotherapy) between the two groups in our study (*p* = 0.508). Furthermore, the median follow‐up time of the study was limited, and long‐term outcomes with the two neoadjuvant regimens remain undetermined. In the future, we will conduct prospective clinical studies based on existing data to further confirm the current conclusions.

In conclusion, in view of the gaps in the current guidelines, we found that patients with initial mrMRF (+) converted to mrMRF (−) after nCT are still at high risk for LRR. For patients with an initial mrMRF (−), nCT alone might be appropriate. In clinical practice, patients with MRF (−) following nCT cannot be treated in the same manner as patients with initial MRF (−). Further prospective studies are required to confirm these findings.

## AUTHOR CONTRIBUTIONS

**Yimin Han:** Data curation (lead); formal analysis (lead); resources (lead); writing – original draft (lead). **Wei xiang Qi:** Methodology (supporting); supervision (supporting); validation (supporting). **Shu bei Wang:** Formal analysis (supporting); software (supporting). **Wei guo Cao:** Conceptualization (supporting). **Jia Yi Chen:** Conceptualization (equal); writing – review and editing (supporting). **Gang Cai:** Conceptualization (equal); writing – review and editing (lead).

## FUNDING INFORMATION

This research received no external funding.

## CONFLICT OF INTEREST STATEMENT

The authors declare no conflict of interest.

## ETHICS STATEMENT

Ethical approval was granted by the Ethics Committee of Ruijin Hospital. This study is a retrospective study, which only collects clinical data of patients and does not interfere with the treatment plan of patients.

## Data Availability

The data that support the findings of this study are available from the corresponding author upon reasonable request.
